# Increased risk of preterm delivery with high cortisol during pregnancy is modified by fetal sex: a cohort study

**DOI:** 10.1186/s12884-022-05061-8

**Published:** 2022-09-23

**Authors:** Brietta M. Oaks, Seth Adu-Afarwuah, Per Ashorn, Anna Lartey, Kevin D. Laugero, Harriet Okronipa, Christine P. Stewart, Kathryn G. Dewey

**Affiliations:** 1 Department of Nutrition and Food Sciences, University of Rhode Island, Kingston, RI 02881 USA; 2 Department of Nutrition and Food Science, University of Ghana, Accra, Ghana; 3 Center for Child, Adolescent and Maternal Health Research, Faculty of Medicine and Health, Technology, Tampere University, Tampere, Finland; 4 Department of Pediatrics, Tampere University Hospital, Tampere, Finland; 5 USDA Western Human Nutrition Research Center, Davis, CA USA; 6 Department of Nutrition, University of California, Davis, CA USA; 7 Department of Nutritional Sciences, Oklahoma State University, Stillwater, OK USA

**Keywords:** Cortisol, Preterm birth, Pregnancy, Fetal sex, Ghana

## Abstract

**Background:**

Previous studies show an association between maternal plasma and salivary cortisol and preterm birth but have been primarily conducted in high-income countries. It is unknown whether salivary cortisol is a risk factor for preterm birth in Ghana. Our objective was to determine whether maternal salivary cortisol during pregnancy was associated with pregnancy duration and preterm delivery in Ghana.

**Methods:**

We conducted a cohort study of 783 pregnant women in Ghana. We measured salivary cortisol at baseline (mean 16 wk), 28 wk., and 36 wk. gestation. Pregnancy duration was determined primarily by ultrasound. We used adjusted linear regression models to examine the association between cortisol and pregnancy duration and Poisson regression models to determine the risk of preterm delivery among women with high cortisol at baseline or 28 wk. gestation.

**Results:**

Mean pregnancy duration was 39.4 ± 1.8 wk. and 6.6% had a preterm delivery. Mean maternal cortisol increased throughout pregnancy, from 4.9 ± 2.7 nmol/L at baseline (16 wk) to 6.4 ± 3.2 nmol/L at 28 wk. and 7.9 ± 3.0 nmol/L at 36 wk. gestation. In adjusted analyses, higher cortisol concentrations at baseline (β = − 0.39, *p* = .002) and 28 wk. (β = − 0.49, *p* = .001), but not 36 wk. (β = − 0.23, *p* = .084) were associated with a shorter pregnancy duration. Women with high cortisol at baseline (> 6.3 nmol/L) had an increased relative risk of preterm delivery (RR (95% CI): 1.96 (1.13, 3.40)), but the association between high cortisol at 28 wk. and preterm delivery was not significant. There was a significant interaction with fetal sex (p-for-interaction = 0.037): among women carrying male fetuses, high cortisol at baseline increased the risk of preterm delivery threefold (3.18 (1.51, 6.71)) while there was no association (1.17 (0.50, 2.74)) among women carrying female fetuses.

**Conclusion:**

Higher maternal cortisol is associated with a shorter pregnancy duration and an increased risk of preterm delivery. Subgroup analysis by fetal sex revealed that this association is evident primarily among women carrying male fetuses. Future studies of cortisol and preterm delivery should include consideration of fetal sex as a potential effect modifier.

**Supplementary Information:**

The online version contains supplementary material available at 10.1186/s12884-022-05061-8.

## Introduction

Preterm birth is the leading cause of child mortality globally, accounting for an estimated 1 million children dying each year from complications that arise from being born too soon [1]. However, the mechanisms that lead to preterm birth, defined as birth before 37 completed weeks of gestation, remain poorly understood and the actual cause of preterm birth is unestablished in most cases [2]. Approximately 81% of preterm births occur in Asia and sub-Saharan Africa, yet the majority of research examining the mechanisms of preterm birth has been conducted in North America and Europe [3, 4].

One potential contributor to premature labor is a high concentration of circulating cortisol. During pregnancy, the placenta produces corticotropin-releasing hormone (CRH), the majority of which enters the maternal bloodstream and stimulates production of cortisol, causing circulating free cortisol to increase, beginning around the 25th week of gestation. The increased maternal cortisol concentration further stimulates additional production of placental CRH, creating a positive feedback loop that leads to higher and higher CRH and cortisol concentrations until birth finally occurs. In addition to stimulating cortisol, CRH also increases placental production of estrogen [5]. While the mechanisms underlying parturition, and thus preterm birth, are still not completely understood, it appears that estrogen plays a key role in this process by upregulating production of oxytocin, a hormone that induces uterine contractions [6]. Most studies [4, 7–9], but not all [10, 11], have shown that women with high cortisol concentrations have a higher risk of preterm, although nearly all of these studies were conducted in high-income countries.

In addition to being a key hormone during pregnancy, cortisol is also part of the hypothalamic-pituitary-adrenal (HPA) axis that responds to stress. Higher cortisol concentrations have been reported among individuals who experience stress from a variety of causes, including food insecurity [12], racism [13], and lower socioeconomic status [14]. Cortisol also responds to physical stressors such as infection [15] or inflammation [16]. Consequently, psychological or physical stress during pregnancy may further increase circulating maternal cortisol concentration, leading to higher concentrations of estrogen and oxytocin too soon in pregnancy, potentially triggering preterm birth [17, 18]. This pathway is supported by other studies showing that women who experience factors such as racism, infection or high-stress events during pregnancy are at higher risk for preterm birth [19–21]. However, other research demonstrates that chronic stress exposure can cause dysregulation of the HPA axis and lead to a blunted cortisol response to stress [22].

The global average preterm birth rate is 10.6%, and in high-income countries the rates are 5–9% [3]. By comparison, Ghana has an estimated preterm birth rate of 12%, resulting in approximately 105,000 infants born preterm in Ghana in 2014 [3]. It is unknown whether high cortisol is a risk factor for preterm births in Ghana. Given that pregnant women in Ghana experience different risk factors for high cortisol than women in high-income countries, including malaria and higher rates of food insecurity, it is possible that they may have a different stress exposure and that the association with preterm birth is not the same as reported in previous studies conducted primarily in high-income countries [4, 7–9, 11]. We have previously reported that among pregnant women in Malawi, higher cortisol during pregnancy was associated with a shorter pregnancy duration, but there was no significant difference in preterm birth rates between women in the highest versus lowest cortisol quartiles [10]. The aim of the current study was to examine the association between cortisol and pregnancy duration in a cohort of pregnant women in Ghana. We hypothesized that higher cortisol would be associated with a shorter pregnancy duration and an increased risk of preterm delivery. Additionally, as previous literature supports maternal age [23], parity [10], and fetal sex [24] as characteristics that may modify associations of risk factors with preterm birth, we also examined whether the association between cortisol and pregnancy duration differed according to these characteristics.

## Methods

### Participants and study design

Women included in this nested cohort study were enrolled in the International Lipid-Based Nutrient Supplements trial (iLiNS-DYAD) in Ghana. Details of the trial study design and primary outcomes have been presented elsewhere [25]. Briefly, women were recruited from antenatal clinics in the Yilo Krobo and Lower Manya Krobo districts, semi-urban areas in the southeast region of Ghana. To be eligible for enrollment, women needed to be ≤20 wk. gestation and ≥ 18 y of age. Women were excluded from the trial if they were HIV positive or had asthma, epilepsy, tuberculosis, a milk or peanut allergy, or a chronic disease that required medical attention. Women who provided signed or thumb-printed informed consent were enrolled from December 2009 to December 2011 and assigned to one of three nutrition supplements taken daily throughout pregnancy: a lipid-based nutrient supplement (LNS), a multiple micronutrient capsule, or an iron-folic acid capsule. Otherwise, the women received normal antenatal care, including intermittent preventive malaria treatment, from their nearest health facility. The Institutional Review Boards of the University of California, Davis; the Noguchi Memorial Institute for Medical Research, University of Ghana; and the Ghana Health Service approved the study protocol, and the trial was registered at clinicaltrials.gov (NCT00970866). Women from the trial were included in the present cohort study if they had at least one measurement of salivary cortisol during pregnancy and an estimated pregnancy duration. We have previously reported that salivary cortisol was similar across the three supplement trial arms, but in a subgroup analysis of women ≤26 years of age, women assigned to the LNS supplement had lower cortisol at 36 wk. gestation [26]. Assigned supplement group was not the focus of the current study and was adjusted for in present analyses.

Pregnancy duration was determined primarily by ultrasound at enrollment, although fundal height was used for a small number of women who were not available for ultrasound. All ultrasounds were performed by doctors at the antenatal clinics using the Aloka SSD 500 Scanner (Tokyo, Japan). A morning saliva sample was collected at enrollment, 28 wk., and 36 wk. gestation. Research nurses collected the saliva samples at enrollment and 36 wk. gestation when participants arrived for their clinic visit and trained data collectors obtained the saliva sample at 28 wk. gestation during a home visit. All saliva samples were collected after at least 30 minutes since eating or drinking and before any other measurements or sample collection. To collect the saliva, women inserted an inert polymer cylindrical swab (10 mm × 30 mm, Salimetrics Oral Swab) under their tongue for approximately 2 minutes and were instructed to move their jaw in a chewing motion to stimulate saliva production. Time of collection, time of waking, and time of last food or drink were recorded. We collected saliva samples between the hours of 8 am and noon, with a mean collection time of approximately 10:30 am. The woman then placed the swab in a capped tube which was then put in a refrigerator or on ice packs. Laboratory technicians brought the swabs to room temperature before centrifuging at 1252 x g for 15 min. All samples were processed and stored at − 20 °C within 24 h of collection and shipped to the USDA Agricultural Research Service Western Human Nutrition Research Center (Davis, CA) for lab analysis. We analyzed the saliva samples in duplicate using Salimetrics Expanded Range High Sensitivity Salivary Cortisol Enzyme Immunoassay Kit (Salimetrics, State College, PA), which can detect cortisol concentrations ranging from 0.193 to 82.8 nmol/L. The intra- and inter-assay coefficients of variation were 4.4 and 7.8%, respectively.

Trained data collectors interviewed women at enrollment about sociodemographic characteristics, household assets, food security, and mode of delivery, and trained anthropometrists measured maternal weight and height and infant birthweight. A phlebotomist collected blood samples by venipuncture at enrollment and 36 wk. gestation and assayed malaria parasitemia with a rapid test (Vision Biotech, South Africa) in peripheral blood. Remaining blood samples were centrifuged at 1252×g for 15 min and plasma samples were stored at − 20 °C and shipped to the USDA ARS Western Human Nutrition Research Center (Davis, CA) where lab technicians measured alpha-1-acid glycoprotein (AGP) and C-reactive protein (CRP) concentrations using a Cobas Integra 400 plus Automatic Analyzer (Roche Diagnostic Corp., Indianapolis, IN). The inter- and intra-assay coefficients of variation for these assays were: AGP intra-assay: < 3%, AGP inter-assay: < 2%; CRP intra-assay: < 4%, CRP inter-assay: < 1%.

### Statistical analysis

We tested variables for normality using the Shapiro–Wilk test and log transformed cortisol, AGP, and CRP. We used linear regression models to examine the association between salivary cortisol concentration (at each of the 3 time points, enrollment, 28 wk. and 36 wk. gestation, as well as the change in cortisol between enrollment and 36 wk) and pregnancy duration, with *p* < 0.05 to indicate statistical significance. Cortisol and pregnancy duration were analyzed as continuous variables and model assumptions were checked using standard regression diagnostics for linearity, leverage, and influence. Because cortisol was log transformed, the decrease in pregnancy duration (wk) was calculated for an x percent increase in cortisol concentration by multiplying the coefficient by log(1.x). Poisson regression was used to estimate relative risk for preterm delivery (< 37 wk. gestation) for women with high cortisol, with *p* < 0.05 to indicate statistical significance. Because there is no established clinical definition of high cortisol during pregnancy, we assessed three common cutoffs used in such situations (highest quartile, highest quintile, top 10%) to examine the association between high cortisol and preterm delivery. If there was a significant association with more than one of those cutoffs, we used the lowest of those cutoffs in order to maximize sample size in the “high cortisol” subgroup. Preterm delivery was examined only with respect to cortisol measured at enrollment and 28 wk. gestation because many preterm births occurred before the 36 wk. saliva sample was collected.

For adjusted analyses, we considered covariates for inclusion in the model if they were significantly (*p* < 0.1) associated with the outcome in bivariate analyses. Based on previous literature, variables identified a priori as potential covariates were gestational age at enrollment, maternal age, parity (nulliparous vs. parous), marital status, education level, household food insecurity, household asset index, season at enrollment (dry vs wet), maternal height, infant sex, twin pregnancy, time since waking, and time since last meal. Household food insecurity was measured using a 9-item scale with responses ranging from 0 to 3. Scores were summed up to obtain a final score which ranged from 0 (no food insecurity) to 27 (every food insecurity condition occurs often) [27]. We created the household asset index based on lighting source, drinking water supply, sanitation facilities, flooring materials, radio, television, refrigerator, cell phone, and stove using principal components analysis [28]. The multivariable models examining 28 wk. and 36 wk. salivary cortisol concentrations were also adjusted for assigned supplement group. Because the inflammatory markers (AGP and CRP) are strongly related to cortisol, but the causal pathway is indeterminate, we conducted adjusted analyses with and without adjustment for AGP and CRP and present both adjusted models. We also conducted a sensitivity analysis with and without twin pregnancies included in the analysis. We identified maternal age, parity, and fetal sex a priori as potential effect modifiers based on previous literature and performed stratified analyses for significant interactions (p-for-interaction < 0.1). All analyses were performed using SAS 9.4 (SAS Institute, Cary, NC).

## Results

**Fig. 1:**
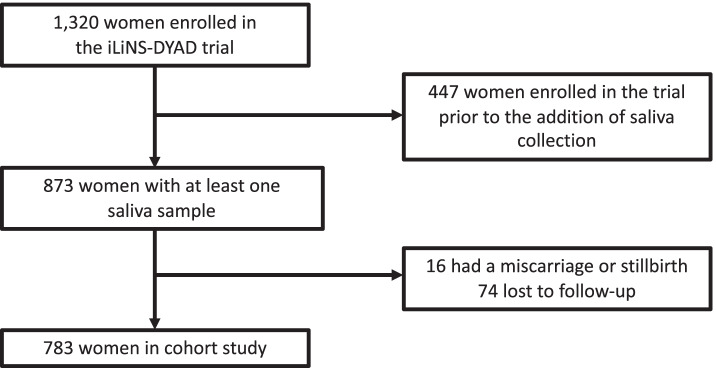
Study participant flow chart

Of the 1320 women enrolled in the trial, 783 women were included in this cohort study (Fig. 1). A single morning saliva sample was collected from 783 women at baseline, 741 women at 28 wk., and 658 women at 36 wk. All women had a baseline saliva sample. Approximately 80.3% had saliva samples at all three timepoints, 13.8% had only the baseline and 28 wk. saliva samples, 3.2% had only the baseline and 36 wk. saliva samples, and 2.7% had only the baseline saliva sample. Included women were similar to excluded women in baseline characteristics (Table 1) and had a mean ± SD age of 26.6 ± 5.4 years, a mean BMI of 24.8 ± 4.6 kg/m^2^, and a malaria prevalence of 9.5%. Mean pregnancy duration was 39.4 ± 1.8 wk. and 6.6% of women had a preterm delivery. Mean maternal cortisol increased throughout pregnancy, from 4.9 ± 2.7 nmol/L at baseline to 6.4 ± 3.2 nmol/L at 28 wk. and 7.9 ± 3.0 nmol/L at 36 wk. gestation. The mean birthweight was 3035 ± 439 g and 17.3% of births were delivered by caesarean section.

**Table 1:** Characteristics of iLiNS-DYAD trial participants at ≤20 weeks gestation who were included in or excluded from the current cohort study

	**Mean ± SD or n/n (%)**		
	Included (***n*** = 783)	Excluded (***n*** = 537)	p
Maternal age (y)	26.6 ± 5.4	26.9 ± 5.7	0.406
Nulliparous (%)	260/783 (33.2%)	186/537 (34.6%)	0.589
Total school years completed (y)	7.6 ± 3.4	7.7 ± 3.9	0.709
Married (%)	720/783 (92.0%)	504/537 (93.9%)	0.263
Gestational age (wk)	16.1 ± 3.0	16.1 ± 3.7	0.851
Male fetus (%)^a^	372/783 (47.5%)	189/376 (50.3%)	0.379
Twin pregnancy (%)^b^	15/783 (1.9%)	7/457 (1.5%)	0.619
BMI (kg/m^2^)	24.8 ± 4.6	24.8 ± 4.5	0.939
Anemia (%, Hb < 100 g/L)	112/783 (14.3%)	73/537 (13.6%)	0.715
Positive malaria test (%)	74/783 (9.5%)	60/536 (11.2%)	0.303
Cortisol, nmol/L	4.9 ± 2.7	4.5 ± 2.2	0.147
AGP, g/L	0.65 ± 0.21	0.65 ± 0.22	0.916
CRP, mg/L	7.2 ± 11.5	6.6 ± 11.7	0.336

^a^Based on infant sex at birth; missing data among excluded women (*n* = 161) due to either miscarriage or loss to follow-up

^b^Based on ultrasound or determined at birth; missing data among excluded women (*n* = 80) due to either miscarriage or loss to follow-up

Overall, a higher salivary cortisol concentration at all three timepoints in pregnancy was associated with a shorter pregnancy duration (Table 2). The association was strongest with cortisol measured at baseline and 28 wk. gestation, with a pregnancy duration that was approximately 1.2 days shorter with a 50% increase in cortisol, after adjusting for covariates. At 36 wk. gestation the association was weaker but still significant, with a pregnancy duration that was 0.8 days shorter with a 50% cortisol increase. When models were additionally adjusted for the inflammatory biomarkers AGP and CRP, the association with cortisol measured at 36 wk. gestation became nonsignificant (*p* = 0.084), but the associations with cortisol measured at baseline and 28 wk. gestation remained significant. Change in cortisol from baseline to 36 wk. was not associated with pregnancy duration.

**Table 2:** Association between pregnancy duration and maternal salivary cortisol (nmol/L) measured at ≤20 wk., 28 wk., and 36 wk. gestation ^a^

Duration of gestation (wk)							
	n (%)	Unadjusted β coefficient (95% CI)	P	Adjusted β coefficient (95% CI), Model 1^b^	p	Adjusted β coefficient (95% CI), Model 2^c^	p
**All women**							
≤ 20 wk. log cortisol	783 (100%)	−0.43 (− 0.66, − 0.20)	<.001	− 0.42 (− 0.66, − 0.17)	0.001	− 0.39 (− 0.64, − 0.14)	0.002
28 wk. log cortisol	741 (94.6%)	−0.43 (− 0.67, − 0.19)	<.001	−0.44 (− 0.73, − 0.15)	0.003	−0.49 (− 0.78, − 0.20)	0.001
36 wk. log cortisol	658 (84.0%)	−0.25 (− 0.49, 0.00)	0.050	−0.29 (− 0.54, − 0.02)	0.032	−0.23 (− 0.49, 0.03)	0.084
Change log cortisol^d^	654 (83.5%)	0.09 (−0.08, 0.27)	0.297	0.08 (−0.11, 0.27)	0.409	0.07 (−0.12, 0.27)	0.447
**Female fetal sex**							
≤ 20 wk. log cortisol	411 (100%)	−0.19 (− 0.51, 0.12)	0.233	− 0.25 (− 0.59, 0.09)	0.151	−0.24 (− 0.59, 0.10)	0.160
28 wk. log cortisol	389 (94.6%)	− 0.37 (− 0.71, − 0.03)	0.031	−0.37 (− 0.77, 0.04)	0.079	−0.37 (− 0.78, 0.04)	0.077
36 wk. log cortisol	338 (82.2%)	0.20 (− 0.11, 0.51)	0.212	0.12 (− 0.21, 0.45)	0.468	0.11 (− 0.22, 0.43)	0.525
Change log cortisol^d^	338 (82.2%)	0.21 (−0.02, 0.44)	0.073	0.19 (−0.05, 0.43)	0.123	0.19 (−0.06, 0.43)	0.129
**Male fetal sex**							
≤ 20 wk. log cortisol	372 (100%)	−0.68 (− 1.02, − 0.34)	<.001	−0.61 (− 0.97, − 0.25)	0.001	−0.57 (− 0.93, − 0.20)	0.003
28 wk. log cortisol	348 (93.5%)	−0.50 (− 0.84, − 0.16)	0.004	−0.44 (− 0.85, − 0.03)	0.037	−0.52 (− 0.93, − 0.10)	0.016
36 wk. log cortisol	316 (84.9%)	−0.82 (− 1.20, − 0.43)	<.001	−0.85 (− 1.26, − 0.44)	<.001	−0.74 (− 1.15, − 0.32)	0.001
Change log cortisol^d^	316 84.9%)	− 0.04 (− 0.32, 0.24)	0.775	−0.04 (− 0.35, 0.27)	0.795	−0.05 (− 0.37, 0.26)	0.752

^a^Associations are presented for log-transformed cortisol concentrations. The decrease in pregnancy duration can be calculated for an x percent increase in cortisol concentration by multiplying the coefficient by log(1.x). E.g., for a 50% increase in cortisol at ≤20 wk. using Model 1, there is a decrease in pregnancy duration by − 0.42*log(1.50) = − 0.17 wk., or − 1.2 days

^b^Model 1 adjusted for supplementation group, gestational age at enrollment, parity, maternal age, education, food insecurity, asset index, BMI, fetal sex, twin pregnancy, time between waking and saliva collection, and time between last food or drink (except water) and saliva collection

^c^Model 2 adjusted for all variables in Model 1, plus AGP and CRP

^d^Change between ≤20 wk. and 36 wk. cortisol concentrations

Of the three cutoffs for high cortisol we examined (top quartile, top quintile, top 10%), the top quintile (above the 80th percentile) and the top 10% for baseline cortisol were both associated with preterm delivery. We used the cutoff of > the 80th percentile (baseline cortisol: > 6.3 nmol/L, 28 wk. cortisol: > 8.5 nmol/L) in subsequent analyses, as explained previously. Women with high cortisol at baseline were twice as likely to have a preterm delivery compared with women below the cutoff of 6.3 nmol/L (adjusted RR (95% CI): 1.96 (1.13, 3.40), *p* = 0.017). This association was not significant at 28 wk. gestation (adjusted RR (95% CI): 1.03 (0.46, 2.29)) (Table 3).

**Table 3:** Risk of preterm delivery for pregnant women with high cortisol during pregnancy

	Cortisol, nmol/L	Cases (%)	Unadjusted RR^a^ (95% CI)	p	Adjusted RR^b^ (95% CI)	p
**All women**						
Baseline	≤6.3	34 (5.5)	1		1	
	> 6.3	18 (11.3)	2.07 (1.21, 3.58)	0.008	1.96 (1.13, 3.40)	0.017
28wk	≤8.5	38 (6.4)	1		1	
	> 8.5	9 (6.5)	1.02 (0.50, 2.06)	0.958	1.03 (0.46, 2.29)	0.946
**Female fetal sex**						
Baseline	≤6.3	21 (6.4)	1		1	
	> 6.3	7 (8.3)	1.30 (0.57, 2.95)	0.534	1.17 (0.50, 2.74)	0.714
28 wk	≤8.5	22 (6.8)	1		1	
	> 8.5	4 (6.0)	0.87 (0.31, 2.45)	0.800	1.16 (0.35, 3.81)	0.807
**Male fetal sex**						
Baseline	≤6.3	13 (4.4)	1		1	
	> 6.3	11 (14.7)	3.35 (1.56, 7.18)	0.002	3.18 (1.51, 6.71)	0.002
28 wk	≤8.5	16 (5.8)	1		1	
	> 8.5	5 (6.9)	1.19 (0.45, 3.16)	0.715	0.86 (0.26, 2.89)	0.812

^a^Relative risk

^b^Adjusted for supplementation group, gestational age at enrollment, parity, maternal age, education, food insecurity, asset index, BMI, fetal sex, twin pregnancy, time between waking and saliva collection, time between last food or drink (except water) and saliva collection, AGP at enrollment, CRP at enrollment

In a sensitivity analysis, results did not differ when women with twin pregnancies were included versus excluded, so both twin and singleton pregnancies were included in the dataset, with twin pregnancy included as a covariate in adjusted analyses.

We tested for interactions and found fetal sex to be a significant effect modifier of the association between cortisol and pregnancy duration (p-for-interaction = 0.037). In adjusted analyses, maternal cortisol at any time point was not significantly associated with pregnancy duration if women were carrying a female fetus, but cortisol was significantly associated with pregnancy duration at all 3 timepoints if women were carrying a male fetus (Table 2). Among women carrying a female fetus, there was no difference in preterm delivery risk with high cortisol concentration. However, among women carrying a male fetus, the risk of preterm delivery was 3 times higher if they had high cortisol at baseline (adjusted RR (95% CI): 3.18 (1.51, 6.71), *p* = 0.002) (Table 3). There was no significant association between preterm delivery and cortisol at 28 wk. gestation among women carrying male fetuses, and no other interactions were significant.

## Discussion

In this cohort study of pregnant women in Ghana, we tested the hypothesis that higher maternal cortisol during pregnancy would be associated with a shorter pregnancy duration and a higher risk of preterm delivery. We found that higher cortisol in early and mid-pregnancy, but not late pregnancy, was associated with a shorter pregnancy duration and high cortisol at baseline was associated with an increased risk of preterm delivery. When this association was further examined by fetal sex, the association was evident only among women carrying male fetuses. If a woman was carrying a female fetus, having high cortisol did not increase her risk of preterm delivery. If a woman was carrying a male fetus, having high cortisol at baseline increased her risk of preterm delivery by threefold. Additionally, we found all associations were maintained even after adjusting for AGP and CRP, supporting higher cortisol as an independent risk factor for shorter pregnancy duration and preterm delivery, regardless of inflammation.

Our results are consistent with numerous studies conducted in high-income countries reporting that high cortisol is associated with preterm birth [4]. However, none of these studies examined whether the association was modified by fetal sex. In our previous study among pregnant women in Malawi, we also showed that higher cortisol was associated with a shorter pregnancy duration (though not preterm birth), but fetal sex was not an effect modifier [10]. Women in the Malawi study had a mean BMI of 22 kg/m^2^, an average of 4 years of completed school, and a malaria prevalence of 22%, which is double the malaria prevalence in the current study. Additionally, although women in the Malawi study were a cohort from a similarly designed nutrition supplement trial that was conducted in parallel to the present study, the Malawi trial included adolescents 15–17 years of age and HIV positive women while the present study did not. Thus, the lack of an effect modification by fetal sex in Malawi may be due to differences between the study populations. For example, malaria during pregnancy and < 18 years of age are both risk factors for preterm delivery [29, 30]. It is possible that with these factors contributing to the risk of preterm delivery in Malawi, the association with cortisol is less prominent and the effect modification by fetal sex is either not present or masked.

While this is the first study to report fetal sex as a modifier of the association between cortisol and preterm delivery, other research is supportive of our results. DiPietro et al. measured maternal salivary cortisol concentrations weekly during pregnancy starting at 24 wk. gestation and found it varied according to fetal sex, with women carrying male fetuses having higher cortisol from 24 to 30 wk. gestation compared to women carrying female fetuses [31]. Additionally, male fetal sex is a risk factor for preterm birth and multiple mechanisms have been proposed to explain this relationship [32]. One potential mechanism relates to the placental 11β hydroxysteroid dehydrogenase (11βHSD-2) enzyme, which converts active cortisol from the maternal bloodstream into its inactive form before it enters fetal circulation. Some research has shown that placental 11βHSD-2 activity is higher in female fetuses, which would result in lower cortisol exposure for female fetuses [33]. Fetal cortisol stimulates the hormonal pathway that results in increased maternal plasma concentrations of estrogen and oxytocin, key hormones involved in the development of contractions. Therefore, if the male fetus has more exposure to cortisol, this could result in a shorter pregnancy duration. This would explain why there was not an association among women carrying female fetuses. If the placentae of female fetuses are better able to deactivate maternal cortisol, high maternal cortisol would have less impact on pregnancy duration among women carrying female fetuses.

Strengths of this study include a large study population with cortisol measured at three different time points during pregnancy. In addition, our study measured a large number and variety of potentially confounding variables, enabling us to better determine the association between cortisol and pregnancy duration by controlling for these variables in adjusted analyses. However, an important limitation is that at each time point in pregnancy, we only have a single measurement of cortisol. The current gold standard is to take several cortisol samples throughout the day at standardized times over the course of 2 days. However, this was not feasible in the present study. Previous studies suggest that one salivary sample collected during the morning is adequate for differentiating women as having high or low cortisol [34]. By not taking multiple measurements of cortisol, our results are biased towards the null, and thus our reported associations may underestimate the magnitude of the association between cortisol and pregnancy duration. Additionally, we do not have data on what proportion of the preterm births were spontaneous versus iatrogenic. Lastly, a small number of women had their gestational age determined by fundal height instead of ultrasound. However, we do not expect these women to be systematically different from the rest of the cohort in terms of cortisol or the risk of preterm birth and their inclusion maintains an analysis that is as reflective of the whole study sample as possible.

## Conclusions

We conclude that high cortisol in early pregnancy increases the risk of preterm delivery in our study setting in Ghana, as is the case in other types of populations. In our cohort, this increased risk of preterm delivery was evident predominantly among women carrying male fetuses, which is a novel finding. As reducing preterm birth has become a critical component of reducing the mortality rate in children under 5 years of age, particularly in lower- and middle-income countries, these results advance our understanding of the association between cortisol and preterm birth. We recommend that future studies on this subject include examination of fetal sex as a potential effect modifier.

## Supplementary Information

**Additional file 1.**

## Data Availability

All data generated or analysed during this study are included in this published article and its supplementary information files.

## Abbreviations

11βHSD-2: 11β hydroxysteroid dehydrogenase
AGP: Alpha-1-acid glycoprotein
BMI: Body mass index
CRP: C-reactive protein
CRH: Corticotropin-releasing hormone
HPA: Hypothalamic-pituitary-adrenal
LNS: Lipid-based nutrient supplement
